# Comparative study of gavage and intraperitoneal administration of gamma-oryzanol in alleviation/attenuation in a rat animal model of renal ischemia/reperfusion-induced injury

**DOI:** 10.22038/IJBMS.2020.51276.11642

**Published:** 2021-02

**Authors:** Yasin Bagheri, Alireza Barati, Sana Nouraei, Nasim Jalili Namini, Mohammad Bakhshi, Ezzatollah Fathi, Soheila Montazersaheb

**Affiliations:** 1 Young Researchers and Elite Club, Tabriz Branch, Islamic Azad University, Tabriz, Iran; 2 Faculty of Veterinary Medicine, Tabriz Branch, Islamic Azad University, Tabriz, Iran; 3 Department of Clinical Sciences, Faculty of Veterinary Medicine, University of Tabriz, Tabriz, Iran; 4 Molecular Medicine Research Center, Tabriz University of Medical Sciences, Tabriz, Iran

**Keywords:** Anti-oxidants, Gamma-oryzanol, Gavage, Intraperitoneal, Renal ischemia/reperfusion, Signaling pathways

## Abstract

**Objective(s):**

Ischemia/reperfusion (I/R) is the leading cause of acute kidney injury. This study aimed to elucidate the reno-protective effect of gamma-oryzanol (GO) by comparing gavage and intraperitoneal (IP) administration methods on renal I/R injury in a rat model.

**Materials and Methods:**

Rats were divided into four groups including (group 1) sham, (group 2) I/R-control, (group 3) I/R+GO gavage-treated, and (group 4) I/R+ GO IP-treated. A single dose of GO was administrated to groups 3 and 4 (100 mg/kg body weight), 60 min before induction of I/R. After anesthesia, I/R was created by 45 min of ischemia, followed by 6 hr of reperfusion. Then, blood and tissue samples were subjected to evaluation of renal function, anti-oxidant capacity, inflammation, apoptotic proteins, and IKB/NF-kB pathway.

**Results:**

The two GO administration methods showed improvement of renal function along with attenuation of histological abnormalities. An increase in antioxidant capacity along with a decrease in pro-inflammatory markers, decline in the expression levels of BAX, Bax/Bcl-2, and caspase-3, and up-regulation of Bcl-2 expression were recorded. Moreover, a significant decrease in NF-Kb, p-IKBα, and MMP-2/9 with an increase in IKBα levels were also observed. Overall, in a comparative evaluation between the two gavage and IP administration methods, we did not find any differences in all examined parameters, except IL-6 which had a better result via gavage.

**Conclusion:**

A single dose of GO administration has a reno-protective effect against renal I/R injury. Gavage and IP administration exhibit similar efficiency in alleviation of I/R injury.

## Introduction

In the kidney, Ischemia/reperfusion (I/R) induced pathological alterations derived acute kidney injury (AKI), is a well-known public health problem worldwide, which is characterized by rapid dysfunction and renal failure (1, 2). I/R injury is a clinical condition caused by blockage of blood supply to a particular organ followed by subsequent restoration of blood flow and re-oxygenation status. In the context of I/R, cellular and molecular events lead to trigger of inflammatory cascades, including the release of reactive oxygen species (ROS), enhancement of cytokine effects, and leukocyte recruitment, which ultimately disturbs the normal function of tissues. In the ischemia phase, the low generation of adenosine triphosphate (ATP) leads to many intracellular abnormalities, such as cell membrane permeabilization, ROS production, and ionic disturbances (3, 4). Reperfusion is the vital stage for supplying oxygen and substrate for ATP synthesis to the ischemic tissue; however, it aggravates tissue impairment through extra generation of ROS (5). This process induces cellular impairment and death. Much growing evidence emphasizes that reperfusion time and re-oxygenation phase is the main contributor to harmful effects in the kidney (6). I/R commonly occurs in many situations such as artery stenosis, sepsis, infarction, and especially during organ transplantation (7). It is shown that apoptosis and inflammatory responses are involved in the pathophysiological events of ischemic AKI (8-10). Interestingly, some evidence shows the beneficial effects of several compounds in combating I/R by decreasing the level of inflammatory cytokines, modulating anti-oxidant activities, and lipid peroxidation (11, 12). ROS production has a crucial role among other mechanisms in cell-induced damage (13). Based on the information above, the administration of anti-oxidants can be a useful therapeutic intervention for alleviating subsequent cell damage via increasing the telomere length (14-16). Recent studies have highlighted the importance of natural compounds in terms of reducing inflammatory reactions as well as the improvement of related complications (17). Gamma-oryzanol (GO) is one of the bioactive components of rice bran oil obtained during the milling process of rice (18). Due to having natural anti-oxidant activity, considerable attention is paid to investigating this compound (19, 20). Many in-line studies have demonstrated that GO has a powerful ability in scavenging free radicals and inhibits lipid peroxidation in oxidative status (21, 22). Anti-inflammatory properties of GO are reported in mice through inhibition of Interleukin 1β (IL-1β), Interleukin 6 (IL-6), and transcription factor NFκB (23, 24). Francisqueti *et al.* (2018) confirmed that GO could ameliorate renal injury in obese animals through modulation of inflammation and oxidative stress markers (25). In another parallel study, Feyzabadi *et al.* (2019) showed that GO can alleviate the toxin-induced oxidative stress in the kidneys of a rat model (26). In addition, it has been reported the GO could ameliorate cardiac ischemia-reperfusion injury through reducing apoptosis events (27). 

Accordingly, we hypothesized that GO, as a known anti-oxidant, may alleviate AKI during I/R injury. To our knowledge, there is no report or evidence concerning the effects of GO in the rat model of renal I/R injury. Therefore, this study aims to elucidate the impact of GO administration on one hand, and in a comparative approach, to evaluate the two gavage and intraperitoneal (IP) administration methods in efficiency improvement as well as alleviation/attenuation of the harmful consequences using renal I/R-induction injury in a rat animal model on the other. 

## Materials and Methods

***Reagents***

GO is a crystalline oil-soluble powder in white or yellowish-white color with a purity of 99%, which was provided as a gift from Tsuno Rice Fine Chemicals Co., Ltd. (Wakayama, Japan). GO at 100 mg/kg/body weight (28) was freshly prepared in 0.5 ml of olive oil on the day of treatment, for both kinds of administration. 

***Experimental animal and study design***

Twenty-four adult male Wistar rats, approximately 3-4 months old (230±20 g), were bought from the Pasture Institute (Tehran, Iran). Before conducting the experiment, the rats were acclimatized to laboratory conditions for ten days. They were housed at 21.00±1.00 °C with a relative humidity of 50.00% and under a photoperiod of 12/12 hr dark/light cycle. They were then fed a standard diet with a constant condition and normal room temperature. The animals were divided into four different groups, each consisting of six rats, including :(1) control group (sham), (2) I/R-control, (3) I/R+GO group treated through gavage administration, (4) I/R+ GO (100 mg/kg body weight) by IP injection. A single dose of GO (100 mg/kg/body weight) was administrated to groups 3 and 4, 60 min before the creation of the ischemic condition(28). All experimental rats were maintained according to the standards of the National Institutes of Health for Laboratory Animal Care and Use.

***Surgical procedure for inducting I/R injury***

After one hour of GO treatment and prior to I/R induction, the rats were anesthetized with xylazine and ketamine at 10 mg/kg and 90 mg/kg (IP), respectively (Bremer Pharma GmbH. Germany). The sham group underwent vascular manipulation, excluding the usage of clamps and, more importantly, without any occlusion. In the other three groups, the surgical procedure was carried out by an experienced surgeon by an abdominal incision, and obstruction of left renal vessels were for 45 min using non-traumatic vascular clamps. Following successful ischemic induction, the color of the kidneys turned pale. Then, the clamps were removed and the abdominal area was sutured and the rats were returned to their cages with free access to food and water. At the end of 6 hr of reperfusion (29), blood samples were taken from the left ventricle of the animal’s hearts under the effect of slight diethyl ether anesthesia for evaluating biochemical parameters. Finally, the rats were euthanized and the left kidney of all experimental groups was taken for subsequent analysis.

***Histopathological examination***

To elucidate the impact of administrated GO on histopathological changes of kidneys, the tissues were collected, fixed by formalin (10% neutral) which was followed by embedding in paraffin. Next, fixed and paraffin-embedded tissues were sectioned, dewaxed, and stained with hematoxylin and eosin (H&E). To examine histopathological alterations, a light microscope (Olympus Corporation, Tokyo, Japan) was used. Histological changes were mainly assessed by measuring the tubular necrosis, via counting the number of necrotic cells, brush border changes, cast formation within the tubules, and tubule dilation were as follows: 0, none; 1, ≤10%; 2, 11–25%; 3, 26–45%; 4, 46–75%; and 5, >76%. The histopathology scoring was done as previously described (30).

***The evaluation of renal function by measuring urea and creatinine***

Renal function was determined by measurement of urea and creatinine (Cr) in the serum samples. Urea and Cr were measured using commercial kits (Pars Azmoon, Iran) with an autoanalyzer (Olympus AU-600, Tokyo, Japan).

***Measurement of MDA as an index of lipid peroxidation***

The effect of GO on lipid peroxidation was evaluated by analyzing MDA levels as a lipid peroxidation marker. The concentration was measured according to the thiobarbituric acid reactive substance (TBARS) and Uchiyama protocol (31). The absorbance was measured spectrophotometrically at 540 nm. 

***Oxidative stress investigation***

To investigate the effect of GO administration on the oxidative status of the experimental groups, the level of enzymatic and non-enzymatic anti-oxidants was evaluated. The levels of superoxide dismutase (SOD), glutathione (GSH), catalase (CAT), and glutathione peroxidase (GP_X_) were determined in I/R induced rats by the colorimetric methods. The SOD activity was measured based on pyrogallol autoxidation, as previously described by Marklund (32). The CAT activity was determined by H_2_O_2_ consumption and measured at 240 nm using the Claiborne method (33). Subsequently, the GSH level was measured and reported based on the previously defined method. Also, serum and renal GPx activities were assessed under a condition similar to our previous study (34).

***Investigation of inflammatory cytokines, apoptosis, and NF-kB protein expression***

Western blotting was used to investigate the expression of apoptosis-related proteins, inflammatory cytokines, pNF-kB/ NF-kB, and p-IkB-α/ IkB-α in all experimental groups. The Western blotting protocol was previously reported by Farahzadi *et al.* (2020) (35). In brief, total protein was extracted from renal tissues using Radio Immunoprecipitation Assay Buffer (RIPA) containing 1% cocktail as a protease inhibitor. Then cell lysate was centrifuged at 12000 rpm for 10 min and protein concentration was calculated with the BCA protein (Pierce Biochemical, Rockford). Then, proteins were separated by sodium dodecyl sulfate-polyacrylamide gel electrophoresis (SDS-PAGE), and then, transferred to polyvinylidene fluoride membrane (EMD Millipore, Billerica, MA, USA). Skim milk was used to prevent nonspecific interaction of proteins in the membrane. After that, the membranes were incubated overnight with primary antibodies against targeted proteins as follow: Bcl-2 associated x protein (BAX) (Santa Cruz Biotechnology, Inc., B-9, Mouse, Monoclonal,sc-7480), B-cell lymphoma 2 (BCL2) (N-19, sc-492 Santa Cruz Biotechnology), caspase 3 (SC-7272, Santa Cruz Biotechnology, Santa Cruz, CA), Cleaved caspase (SC-56052-Santa Cruz, CA), IL1-B (sc-32294, Santa Cruz Biotechnology), IL-6 (E-4; sc-28343; dil. 1:1000; Santa Cruz Biotechnology, Santa Cruz, CA, USA), tumor necrosis factor-alpha** (**TNF-α) (4E1]:sc-130349 Santa Cruz biotechnology, In), p- NFkB (Abcam, No: ab16502, Dilution: 1:250), p-IKB1 (Elabscience Biotechnology), MMP2 (H-76; mouse monoclonal antibody against human MMP-2, sc-10736, Santa Cruz Biotechnology, USA) and β-actin (1: 10,000, sc-47778, Santa Cruz Biotechnology Inc). After that, following washing with Tris-buffered Saline-Tween 20 (TBST) 3 times for 5 min , the membranes were subsequently stained with secondary antibodies (goat anti-mouse IgG-HRP: SC-2031) at room temperature for 2 hr. Finally, the membranes were washed and protein bands were detected using an enhanced chemiluminescence detection kit (Roche, UK) with X-ray film. The intensity of protein bands was measured using the ImageJ 1.6 software package and signal intensity of each band was normalized to its corresponding β-actin control (36, 37).

***Statistical analysis***

The obtained results were expressed as mean± SEM. To analyze data, we used one-way analysis of variance (ANOVA) followed by *post hoc* Tukey’s tests. IBM SPSS 16.0 Software package (SPSS, Inc.) was used for statistical analysis. *P-value*<0.05 was considered statistically significant. All experimental procedure was repeated three times.

## Results

***GO administration attenuates renal I/R induced histological abnormalities***

**Figure 1:**
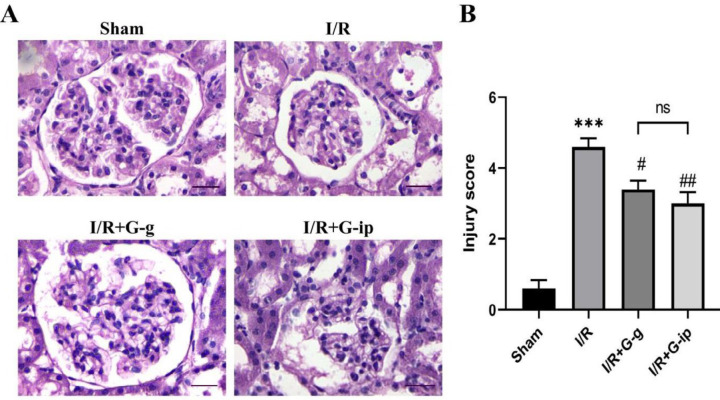
Histopathological examination of I/R-induced renal injury. H & E staining (400 ×) of kidney sections in the sham group, I/R, IR + G-g, and IR + G-ip animals are depicted (A). ***P<0.001 compared with the sham group; # *P*<0.05 and ##*P*<0.01 compared with the I/R induced rats (B). H&E: hematoxylin and eosin; I/R: ischemia-reperfusion, ns: non-significant (Scale bar=25 μm)

The histological alterations of all experimental groups are shown in Figure 1A. Following renal I/R injury, tubule atrophy and necrosis were observed in the I/R group when compared with the sham group (*P*<0.001). However, the morphological abnormalities were significantly attenuated in the GO treated groups by either gavage or IP administration as compared with the I/R group (*P*<0.05, *P*<0.01; respectively) (Figure 1B). These results imply that a single dose of GO treatment attenuates I/Rinduced morphological abnormalities.

***The serum levels of creatinine (Cr) and urea decreased in I/R treated rats***

To evaluate renal function, levels of Cr, urea, and protein were determined in serum samples of all experimental groups as functional parameters. As depicted in Table 1, I/R injury led to a substantial rise in the levels of serum urea and Cr along with a significant reduction of protein levels in comparison with the sham group (*P*<0.001, *P*<0.01, and *P*<0.01, respectively), highlighting I/R- mediated dysfunction in kidneys. Following GO administration, a considerable decrease was observed in the serum levels of urea by gavage and IP injection as compared with I/R control (*P*<0.001 and *P*<0.01). Besides, the level of serum Cr significantly diminished in treatment by both gavage and IP in comparison with the untreated I/R group (*P*<0.01 and *P*<0.01). Moreover, the level of total protein slightly increased in gavage and IP administration compared with the untreated I/R group. It is noteworthy that GO at 100 mg/kg was sufficient to reduce urea and Cr concentrations in the I/R group to the average level in the sham group. No significant difference was observed between the two kinds of administration (*P*>0.05).

***The effect of GO on oxidative activity of I/R rats***

**Figure 2:**
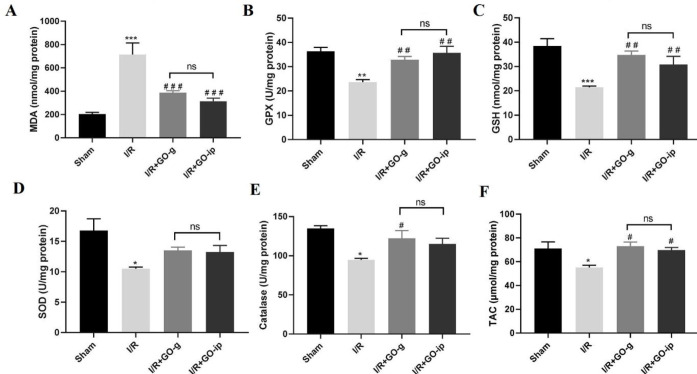
Effect of GO on anti-oxidant capacity: MDA levels (A); GPX activity (B); GSH activity(C); SOD activity (D); CAT activity (E); TAC levels (F). All values are mean±SEM. * *P*<0.05, ** *P*<0.01, and *** *P*<0.001 compared with sham control; # *P*<0.05, ## *P*<0.01, and ###*P*<0.001 compared with I/R. ns: non-significant

As shown in Figure 2, renal I/R injury caused a dramatic increase in MDA levels (nmol/mg protein) as compared with the sham group (*P*<0.001), whereas GO treatment with two kinds of administration, provided a significant decline in MDA levels in I/R-induced rats (*P*<0.001) (Figure 2A). Also, I/R injury resulted in a drastic reduction of anti-oxidant capacity of GPX, GSH, SOD, CAT, and total anti-oxidant capacity (TAC) in the kidneys of I/R rats when compared with the sham group (*P*<0.01, *P*<0.001, *P*<0.05, *P*<0.05, and *P*<0.05, respectively). Administration of GO improved the activity of these enzymes in the kidney tissues (Figures 2B, C, D, E, and F). For both GPX and GSH, a substantial increment was observed by both IP injection and oral gavage (*P*<0.01) (Figures 2 B and C). Furthermore, a significant increase in the activity of CAT (*P*<0.05) was observed only by oral gavage (Figure 2E). However, the SOD levels did not show any significant alteration in both kinds of administration (Figure 2D). The results also revealed a significant increase of TAC in treated groups both by gavage and IP injection (P<0.05) (). These findings did not show any significant change between gavage and IP administration (*P*>0.05). These data implied that GO treatment could improve the activity of anti-oxidant enzymes as ROS scavenge in IR injury. 

***Pro-inflammatory cytokines reduced in I/R rats treated with GO***

**Figure 3:**
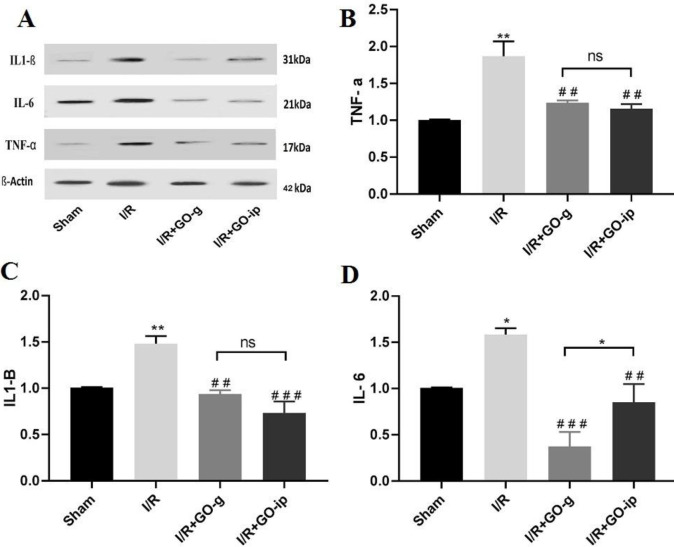
GO attenuates I/R induced alterations in TNF α, IL 1β, and IL 6 expression levels. Representative images of Western blot results for inflammatory markers (A). The levels of TNF-α (B), IL-1β (C), and IL-6(D) in renal tissues of rats. * *P*<0.05 and ** *P*<0.01 compared with sham control. ## *P*<0.01 and ###*P*<0.001 compared with I/R. ns: non-significant. All values are mean±SEM

To examine whether I/R injury may have an impact on inflammation, the expression level of pro-inflammatory cytokines was analyzed by Western blotting in all experimental groups. As depicted in Figure 3, compared with the sham group, the expressions of TNF-α, IL-1β, and IL-6 were significantly induced in I/R injury (*P*<0.01, *P*<0.01, and *P*<0.05, respectively). Following GO administration through gavage, the expression levels of TNF-α, IL-1β, and IL-6 significantly reduced in I/R treated groups (*P*<0.01, *P*<0.01, and *P*<0.001, respectively) compared with those in the I/R group. Besides, IP injected rats had a reduction in the levels of pro-inflammatory cytokines (*P*<0.01, *P*<0.001, and *P*<0.01, respectively) when compared with the I/R group (Figures 3B, C, and D). These results indicate that GO administration attenuates renal IRmediated inflammatory processes. No significant difference was detected between two routes of administration regarding TNFα and IL1β (*P*>0.05), however; gavage administration had a better reduction in the level of IL-6 when compared with IP injection (*P*<0.05).

***The effects of GO administration on the expression of apoptosis-associated factors in renal I/R injury***

**Figure 4:**
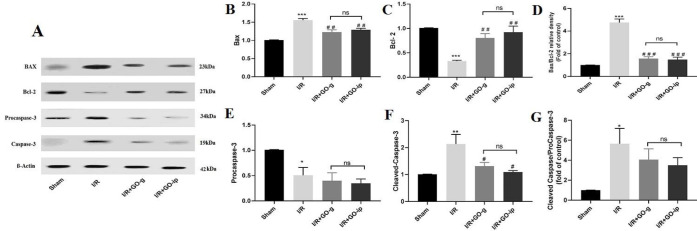
GO protected against I/R induced activation of caspase 3 and BAX expression. Representative images of Western blot results for apoptotic proteins (A). The levels of Bax (B), Bcl-2 (C), and Bax/Bcl-2(D), procaspase-3(E), cleaved caspase-3(F) and cleaved caspase 3/ procaspase-3 (G) in renal tissues of rats. * P<0.05, and *** P<0.001 compared with sham control; # P<0.05, ## P<0.01, and ### P<0.001 compared with I/R. ns: non-significant. All values are mean± SEM

To investigate the impact of GO on pro-apoptotic and anti-apoptotic proteins, the Western blotting analysis was performed in all experimental groups. Compared with the sham group, the expression levels of Bax and cleaved caspase-3 were up-regulated (*P*<0.001, *P*<0.01) (Figures 4B and F), whereas the expression of Bcl-2 and procaspase-3 decreased in I/R group rats (*P*<0.001, *P*<0.05) (Figures 4 C and E). Following administration of GO both by gavage and IP routes, the protein levels of Bax (*P*<0.01, *P*<0.01) and cleaved caspase-3 (*P*<0.5, *P*<0.05) significantly declined in I/R treated rats and the protein level of Bcl-2 was remarkably increased (*P*<0.01, *P*<0.01) in comparison with the untreated I/R rats. In the case of comparative assessment of two routes of administration, no significant differences were detected in the expression level of apoptotic- proteins (*P*>0.05). The results presented here indicate that GO treatment decreases the expression level of Bax and cleaved caspase-3 and up-regulates the expression of Bcl-2 in renal IR injury.

***Effect of GO on IKB/NF-*** ***κB*** ***signaling pathway in renal I/R injury***

**Figure 5:**
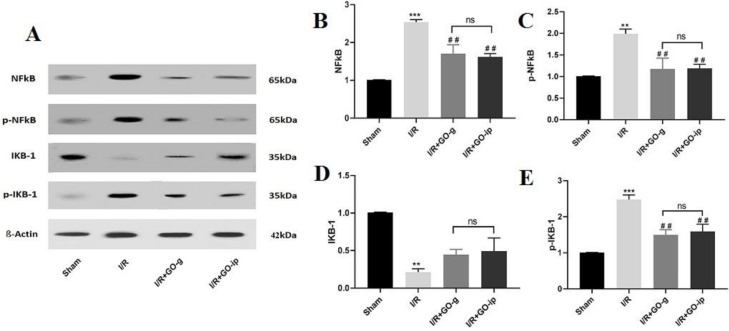
Effect of GO on p-NF-κB/NF-κB and p-IKBα/IKBα signaling in rats with I/R. Representative images of Western blot results (A). The levels of NF-κB (B), p-NF-κB (C), IKBα (D), and p-IKBα in renal tissues of rats. **P<0.01 and *** *P*<0.001 compared with sham control; ## *P*<0.01 compared with I/R. ns: non-significant. All values are mean±SEM

To evaluate whether GO is involved in activation of NF-κB signaling in the rats undergone I/R injury, the protein levels of p-IκBα/IκBα and p-NF-κB/NF-κB were determined by Western blotting. The results showed that the expression levels of NF-κB, p-NF-κB, and p-IκB-α proteins significantly increased in I/R rats (*P*<0.001, *P*<0.01, and *P*<0.001, respectively) (Figures 5B, C, and E), whereas the level of IκB-α significantly decreased when compared with those in the sham group (*P*<0.01) (Figure 5D). However, treatment with GO resulted in a significant reduction in the expression levels of NF-κB, p-NF-κB, and p-IκB-α proteins in I/R treated rats with two kinds of administration routes (*P*<0.01 and *P*<0.01) when compared with those in the untreated I/R rats. The expression level of IκB-α did not change following GO treatment in both administration routes (*P*>0.05) (Figure 5D). 

***Effect of GO on matrix metalloproteinase (MMPs) protein in renal I/R injury***

**Figure 6:**
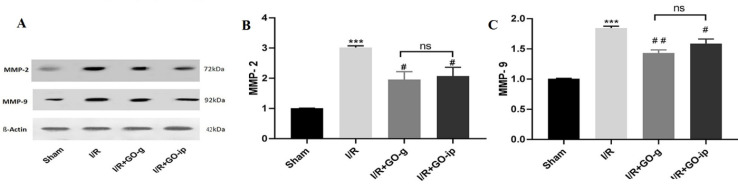
Effect of GO on MMP-2 and MMP-9 protein expression in rats with I/R. Representative images of Western blot results of MMP-2 and MMP-9. The levels of MMP-2 (B) and MMP-9 (C)in renal tissues of rats. ***P<0.001 compared with sham control, ## *P*<0.01 compared with I/R. All values are mean±SEM (n=3)

Western blot analysis was conducted in order to quantify the MMPs protein level in all experimental groups. As indicated in Figure 6 (B and C), Western blot analysis revealed that expression levels of MMP-2 and MMP-9 proteins had a higher level in the I/R rats (*P*<0.001, *P*<0.001) as compared with the sham group. Following GO treatment of the I/R rats, a significant reduction in the expression level of MMP-2 (*P*<0.05) was observed with both administration routes (Figure 6B). The MMP-9 expression level was reduced dramatically in I/R rats treated with both kinds of administration (*P*<0.01, *P*<0.05) (Figure 6C). 

## Discussion

This comparative study was evaluated by comparing the two gavage and IP injection of GO administration methods to investigate their effects on renal function and histology, oxidative stress, inflammatory markers, IKB/NF-Κb signaling pathway, and MMPs expression level in rats induced by renal I/R.

Kidneys excrete urea and Cr as a waste metabolic product, hence, the plasma levels of these parameters can reflect renal function. In this study, the levels of serum Cr and urea of I/R rats were higher than those in the sham-operated groups. Based on our hypothesis and the current results, the I/R-induced rats had functional disturbances of the kidney that occurred after the I/R operation. Interestingly, GO administration was able to reduce the concentrations of urea, Cr, and total protein, indicating the reno-protective effect of this compound. These effects may be related to anti-inflammatory and anti-oxidant activity of the GO substance. These findings are in accordance with previously published reports regarding the nephroprotective effect of the naturally-derived product (38). Comparative assessment of two routes of administration revealed similar efficiency improvement of the renal function. 

Several studies have reported that some plants with anti-oxidant properties can attenuate renal injury resulting from oxidative stress during I/R status (39-42). Therefore, anti-oxidants are often used as a therapeutic option to protect cells from free radicals and oxidative damage (43, 44). In accordance with these findings, the results of this study showed that I/R-induced rats had low levels of GPx, GSH, CAT, SOD, and TAC, but an increased level of MDA expression, indicating that I/R rats experienced oxidative stress. Our results indicated that following GO treatment, the activities of GPx, GSH, CAT, SOD, and TAC were increased, and the level of MDA was decreased in I/R rats. In fact, changes in anti-oxidant activities and MDA content are important indices of oxidative stress (45). Therefore, as a possible consequence of oryzanol therapy, it can be inferred that the increase in anti-oxidant capacity may be attributed to the diminished level of ROS production. The results are in line with the previous observation (46). Noteworthy, the results of gavage and IP administration were at equal values, implying that route of administration did not alter the effectiveness of the treatment.

 The inflammatory response indicates the biological defense of the immune system to restore the original status caused by an injury. Based on the potential of I/R injury in the induction of a sequel of inflammatory events (47), the determination of TNF-α, IL-1, and IL-6 levels was conducted as an important index in the inflammatory state of the body. According to the obtained results, I/R-induced rats had a higher level of pro-inflammatory cytokines. In the presence of GO, a considerable decline was observed in the level of pro-inflammatory markers in both types of administration in I/R-induced rats, however, in the case of IL-6, gavage administration showed a better result. These findings are consistent with previously published reports concerning the effects of plant-derived products on inflammatory responses (48, 49).

 The apoptotic pathway relies on caspase enzymes, as essential mediators in renal I/R injury (50). Caspase-3 not only activates the induction of apoptosis but also triggers inflammatory responses after I/R injury in animal models (51). It is believed that induction is the main contributor to renal I/R injury (52). Besides, it has been shown that ROS production not only inactivates anti-oxidant enzymes but can also contribute to the activation of apoptotic genes as well as caspase cascades (53). It is well documented that Bax induces a triggering pathway, whereas Bcl-2 protein behaves controversially and prevents caspase activation and ROS production. Also, caspase-3 can be involved in the reduction of Bcl-2 and promotion of Bax expression (9, 54). In keeping with this conception, to investigate whether GO treatment could cause an attenuation/alleviation effect on apoptosis induction, the expression levels of apoptotic-related proteins were examined. The findings showed that Bax and caspase-3 protein levels were up-regulated, while Bcl-2 was decreased in I/R injury rats. Indeed, GO administration could dramatically inhibit the I/Rinduced activation of caspase-3 and Bax expression in rats exposed to the renal I/R injury accompanied by increased level of Bcl-2 level in I/R injury models. It is worth mentioning that comparison of both gavage and IP administration showed similar efficiency in the apoptotic-mediated pathway. Considering that apoptosis and inflammation are central contributors to organ damage, the findings of this project will provide a new view and evidence for the protective effect of GO therapy in the case of renal I/R injury. 

NF-κB is considered a critical mediator in regulating inflammatory cytokines, hence, it was hypothesized that inhibition of the NF-κB pathway might decrease inflammatory responses. Based on this information, we elucidated the effect of GO therapy in IKB/NF-Κb. Besides, it has been shown that NF-κB serves a vital role in inflammation, thus, inhibition of this protein may reduce systemic inflammation (55). The results of the present study revealed that renal tissues of the model groups had higher levels of NF-κB p-65 and p-IKBα/IKBα content than the sham group, indicating that renal tissues of the I/R-induced rats had inflammatory status via activation of the inflammation-related signaling pathway. Treatment with GO markedly reduced the expression level of the NFκB protein. Importantly, the comparison of the two methods of GO administration showed similar effectiveness. Overall, GO showed potential as a reno-protective agent against I/R injury. These results are in agreement with other studies regarding the attenuation of oxidative stress and inflammation in I/R-induced rats through inhibition of NF-κB signaling (56, 57). We then investigated whether the increased level of pro-inflammatory cytokines in I/R rats had an effect on metalloproteinase activation such as MMP-2 and MMP-9. Previous study has shown the activities of MMP-2 and MMP-9 in renal tissue of I/R injury and it has been proven that there is a direct correlation between the levels of these metalloproteinases and the severity of inflammation (58). Consistent with these observations, the findings of the present study showed higher levels of MMP-2 and MMP-9 in rats who underwent the ischemic-reperfusion procedure. However, GO administrations ameliorated the expression levels of MMP-2 and MMP9, manifesting the ability of GO therapy in down-regulation of MMP-2 and MMP-9. Although a variety of reasons have been proposed for underlying mechanisms, attenuation of inflammatory markers may be considered in I/Rtreated rats. Of note, comparison of the two methods of GO administration showed equality on the expression level of MMP-2/9.

**Table 1:** Effect of gamma-oryzanol on the level of serum creatinine, urea, and total protein in experimental rats

	Urea (mg/dl)	Creatinin (mg/dl)	Total protein (g/dl)
Sham	68.75± 3.81	0.78± 0.04	6.36± 0.21
I/R	110.50± 3.12 ***	1.13± 0.05 **	5.34± 0.09 **
I/R+ Gg	77.00± 4.18 ###	0.74± 0.06 ##	5.92± 0.23
I/R + G-ip	87.75± 3.32 ##	0.85± 0.05 ##	5.72± 0.11

Effect of two kinds of gamma-oryzanol administration on renal function. **P<0.01 and *** *P*<0.001 compared with sham control; ##*P*<0.01 and ###*P*<0.001 compared with I/R

## Conclusion

Based on the present findings, it can be concluded that a single dose of GO administration may have a beneficial and reno-protective effect on renal I/R injury. These effects of GO are probably mediated through attenuating and interfering with a series of events, including inflammation and apoptosis pathways in renal tissues. Comparative analysis between gavage and IP administration of GO, exhibits almost similar effectiveness in alleviation of I/R injury, highlighting the potential of GO rather than administration routes.

## Data Availability

The data sets used and/or analyzed during the current study are available from the corresponding author on reasonable request.
